# Ecosystem Services, Physiology, and Biofuels Recalcitrance of Poplars Grown for Landfill Phytoremediation

**DOI:** 10.3390/plants9101357

**Published:** 2020-10-14

**Authors:** Ronald S. Zalesny Jr., J. Y. Zhu, William L. Headlee, Roland Gleisner, Andrej Pilipović, Joris Van Acker, Edmund O. Bauer, Bruce A. Birr, Adam H. Wiese

**Affiliations:** 1 USDA Forest Service, Northern Research Station, Rhinelander, WI 54501, USA; ebauer@charter.net (E.O.B.); bbirr@centurytel.net (B.A.B.); adam.wiese@usda.gov (A.H.W.); 2 USDA Forest Service, Forest Products Laboratory, Madison, WI 53726, USA; junyong.zhu@usda.gov (J.Y.Z.); roland.gleisner@usda.gov (R.G.); 3 Weyerhaeuser Company, Hot Springs, AR 71901, USA; bill.headlee@weyerhaeuser.com; 4 Institute of Lowland Forestry and Environment, University of Novi Sad, 21102 Novi Sad, Serbia; andrejp@uns.ac.rs; 5 Laboratory of Wood Technology (UGent-Woodlab), Ghent University, B-9000 Ghent, Belgium; joris.vanacker@ugent.be

**Keywords:** biomass productivity, carbon isotope discrimination (Δ), carbon storage, glucose yield, phytoaccumulation, phytoextraction, phytotechnologies, *Populus*, substrate enzymatic digestibility (SED), wood composition

## Abstract

Long-term poplar phytoremediation data are lacking, especially for ecosystem services throughout rotations. We tested for rotation-age differences in biomass productivity and carbon storage of clones *Populus deltoides* Bartr. ex Marsh × *P. nigra* L. ‘DN34′ and *P. nigra* × *P. maximowiczii* A. Henry ‘NM6′ grown for landfill phytoremediation in Rhinelander, WI, USA (45.6° N, 89.4° W). We evaluated tree height and diameter, carbon isotope discrimination (Δ), and phytoaccumulation and phytoextraction of carbon, nitrogen, and inorganic pollutants in leaves, boles, and branches. We measured specific gravity and fiber composition, and determined biofuels recalcitrance of the Rhinelander landfill trees versus these genotypes that were grown for biomass production on an agricultural site in Escanaba, MI, USA (45.8° N, 87.2° W). ‘NM6′ exhibited 3.4 times greater biomass productivity and carbon storage than ‘DN34′, yet both of the clones had similar Δ, which differed for tree age rather than genotype. Phytoaccumulation and phytoextraction were clone- and tissue-specific. ‘DN34′ generally had higher pollutant concentrations. Across contaminants, stand-level mean annual uptake was 28 to 657% greater for ‘NM6′, which indicated its phytoremediation superiority. Site-related factors (not genotypic effects) governed bioconversion potential. Rhinelander phytoremediation trees exhibited 15% greater lignin than Escanaba biomass trees, contributing to 46% lower glucose yield for Rhinelander trees.

## 1. Introduction

Covering more than 31-million hectares across 30 countries [1], poplar biomass plantings are one of the most widespread, fast-growing feedstock production systems worldwide [2]. In addition to biomass production for biofuels, bioenergy, and bioproducts [3], poplars and their hybrids are ideal for phytoremediation [4]. Large root systems, fast growth, and elevated transpiration rates, combined with the availability of specially-bred, vegetatively-propagated material, make poplars model candidates for phytotechnologies [5,6,7]. In particular, poplars have been shown to accumulate heavy metals and other inorganic pollutants [8,9,10] and metabolize organic contaminants and excessive nutrients [11,12,13] in soils and groundwater. Poplars are commonly used in landfill applications and they have been effective in decreasing contamination through processes, such as phytoextraction, phytostabilization, and runoff reduction [7,14,15,16].

Regardless of end use, genotype × environment interactions play a substantial role in poplar establishment and subsequent plantation development [17,18]. This is especially true for phytotechnologies, where genotypic responses not only depend on climatic and edaphic factors, but also conditions that are imposed by pollutants of the system (e.g., contaminated soils, wastewater, etc.). In addition to provisioning ecosystem services, such biomass and freshwater [19], poplars used in phytoremediation provide additional ecosystem services [7,20]. For example, supporting services, such as nitrogen and water cycles, are tightly linked to regulating services, such as carbon sequestration and water quality, both of which phytotechnologies provide [21]. Poplars are among the most productive temperate-grown trees, with global average biomass mean annual increment (BIOMASS_MAI_) of 11.2 Mg ha^−1^ yr^−1^ and North American average of 12.3 Mg ha^−1^ yr^−1^ [22]. Carbon sequestration potential of poplars is also relatively higher than other temperate genera, with an average of 47% of poplar biomass being carbon [7]. Predictive modeling of poplars in the Southeast United States resulted in carbon mean annual increment (CARBON_MAI_) values of 9.9 Mg C ha^−1^ over a 100-year rotation [20], while poplar plantations in the North Central United States have shown CARBON_MAI_ as high as 10.2 Mg C ha^−1^ [23].

Overall, the majority of tree-based phytoremediation studies have focused on efficiency (e.g., accumulation and degradation of pollutants) and performance (e.g., tree survival, growth, and physiological responses) of trees grown in the presence of contaminants [2,24]. Therefore, directly quantifying biomass and carbon ecosystem services is essential for gauging phytoremediation success. Other ecosystem services are equally important. For example, poplars exhibit phreatophytic characteristics, such as high water uptake and transpiration [25,26,27] and the ability to economize water use in moisture limited areas [28]. Thus, water use efficiency (WUE), which is strongly correlated with both δ^13^C stable carbon isotope ratios and carbon isotope discrimination (Δ) [29,30], is an important trait during genotype selection for phytoremediation. However, research using Δ in phytoremediation with trees is scarce [31], especially when considering potential stress impacts that are caused by landfill soil properties [32,33] and/or highly variable contamination sources [10,34,35] on plant water regimes during phytoremediation processes.

In addition to measuring the efficiency and performance of ecosystem services and physiological traits, quantifying biofuels recalcitrance incorporates an aspect of phytoremediation that is not often considered: end use of the biomass feedstock [5,36]. Converting poplar biomass into biofuels, bioenergy, and bioproducts has been the focus of much research [3,37,38], and promising pretreatment methods have been developed and tested on poplars during the last decade [38,39,40]. In particular, dilute acid (DA) and sulfite pretreatment for overcoming the recalcitrance of lignocelluloses (SPORL) pretreatments were used to compare biofuels recalcitrance of native quaking aspen (*Populus tremuloides* Michx.) with three poplar clones (*Populus deltoides* Bartr. ex Marsh × *P. nigra* L. ‘NE222′ ‘DN5′ and *P. nigra* × *P. maximowiczii* A. Henry ‘NM6′). ‘NE222′ and ‘DN5′ both exhibited bioconversion potential intermediate of pretreated quaking aspen and ‘NM6′, indicating potential for these methods given that ‘NE222′ and ‘DN5′ have six to eight times greater biomass productivity than quaking aspen along with 36 to 71% greater ethanol yield than ‘NM6′, depending on the pretreatment method [41].

There is a lack of long-term data from poplar installations, despite being used more than any other woody genera for phytoremediation in temperate regions [42]. In particular, there is a knowledge gap about the diversity and magnitude of ecosystem services poplars provide from crown closure to the end of their rotations [7]. To learn about these long-term benefits, we tested for rotation-age differences in biomass productivity and the carbon storage potential of clones *Populus deltoides* Bartr. ex Marsh × *P. nigra* L. ‘DN34′ and *P. nigra* × *P. maximowiczii* A. Henry ‘NM6′ used for landfill phytoremediation in Rhinelander, WI, USA (45.6° N, 89.4° W). In addition to these ecosystem services, we evaluated tree height and diameter, carbon isotope discrimination (Δ), and phytoaccumulation and phytoextraction of carbon, nitrogen, and inorganic pollutants (Al, Cd, Ca, Co, Cr, Cu, Fe, K, Mg, Mn, Na, Ni, P, Pb, Zn) in leaves, boles, and branches. We measured specific gravity and fiber composition and determined biofuel recalcitrance of the Rhinelander landfill trees versus these genotypes that were grown for biomass production on an agricultural site in Escanaba, MI, USA (45.8° N, −87.2° W). Overall, these data are important for resource managers, researchers, and regulators that are designing, implementing, and monitoring phytoremediation and associated phytotechnologies at landfills and similar liability sites. In addition, biofuel conversion information is useful for evaluating potential biomass feedstock sources from non-traditional applications, thus reducing pressure on purpose-grown plantations and natural forests [36].

## 2. Results

### 2.1. Ecosystem Services and Physiology

**Figure 1:**
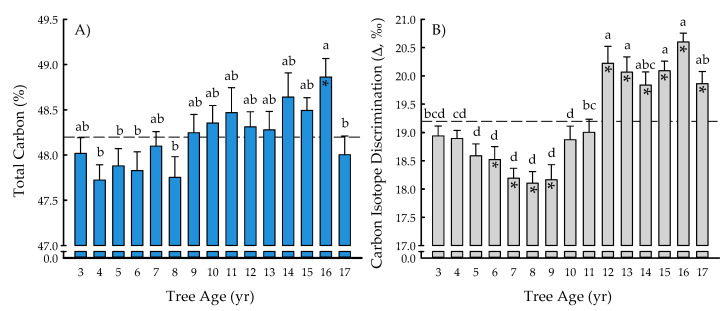
Total carbon (%) (**A**) and carbon isotope discrimination (Δ, ‰) (**B**) from tree age 3 to 17 years across two hybrid poplar clones [*Populus deltoides* Bart. ex Marsh. × *P. nigra* L. ‘DN34′; *P. nigra* × *P. maximowiczii* A. Henry ‘NM6′] grown for phytoremediation at the former Rhinelander City Landfill in Rhinelander, WI, USA. The dashed line is the overall mean; means differing from the overall mean at *p* < 0.05 are indicated with asterisks. Bars with the same letters are not different at *p* < 0.05.

The tree age main effect was significant for total carbon (*p* = 0.0023) and carbon isotope discrimination *(p* < 0.0001) (Table 1). The total carbon ranged from 47.72 ± 0.17% (four years) to 48.86 ± 0.21% (16 years), with an overall mean of 48.20 ± 0.05% (Figure 1A). In general, the total carbon increased over time until 16 years after planting, then significantly decreased 2% during the last year of the study. Carbon content of 16-year-old trees was also 2% greater than that of 4-, 5-, 6-, and 8-year-old trees. Carbon isotope discrimination (Δ) ranged from 18.10 ± 0.20‰ (eight years) to 20.60 ± 0.16‰ (16 years), with an overall mean of 19.20 ± 0.08‰ (Figure 1B). From years six to nine, Δ decreased 2 to 4% as compared with three-year-old trees, with Δ during those four years being 4 to 6% significantly less than the overall mean. At years 10 and 11, Δ increased until reaching its maximum at 16 years, which was not different than at 12, 13, 14, 15, or 17 years. Values for Δ during the last six years of the study were 3 to 7% greater than the overall mean (Figure 1B).

**Figure 2:**
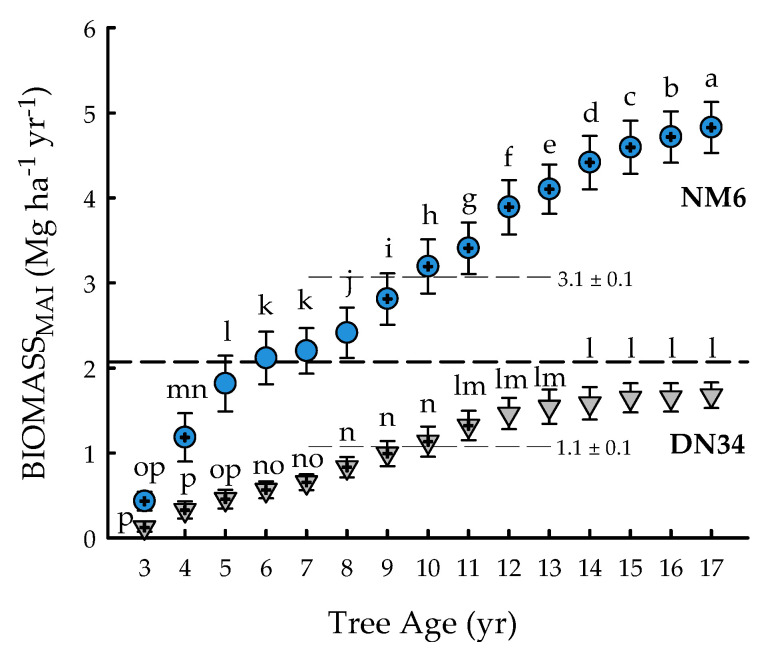
Mean annual increment of aboveground dry leafless biomass (BIOMASS_MAI_; Mg ha^−1^ yr^−1^) from tree age 3 to 17 years for two hybrid poplar clones [*Populus deltoides* Bart. ex Marsh. × *P. nigra* L. ‘DN34′; *P. nigra* × *P. maximowiczii* A. Henry ‘NM6′] grown for phytoremediation at the former Rhinelander City Landfill in Rhinelander, WI, USA. The dashed line is the overall mean; means differing from the overall mean at *p* < 0.05 are indicated with plus symbols. Values for clonal means are shown. Triangles (‘DN34′) and circles (‘NM6′) with the same letters are not different at *p* < 0.05.

The clone × age interaction was significant for BIOMASS_MAI_ (*p* < 0.0001) (Table 1). BIOMASS_MAI_ ranged from 0.1 ± 0.1 (‘DN34′, three years) to 4.8 ± 0.3 (‘NM6′, 17 years) Mg ha^−1^ yr^−1^, with an overall mean of 2.1 ± 0.1 Mg ha^−1^ yr^−1^ (Figure 2). The annual biomass production of ‘DN34′ stabilized during years 14 to 17, while that of ‘NM6′ continued to increase until harvest at age 17 years. Overall, ‘NM6′ exhibited 182% greater BIOMASS_MAI_, averaged across the rotation. BIOMASS_MAI_ of ‘DN34′ never reached the overall mean, and annual productivity for ‘DN34′ from years three to 11 was 36 to 94% significantly less than the overall mean. In contrast, BIOMASS_MAI_ of ‘NM6′ was 36 to 133% significantly greater than the overall mean from years 9 to 17 (Figure 2).

**Figure 3:**
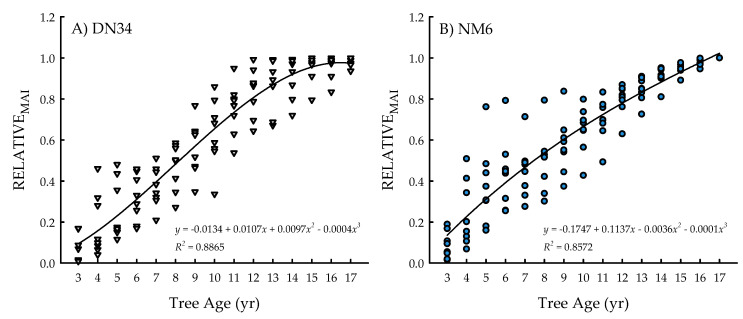
Relative mean annual increment (RELATIVE_MAI_; proportion of current-year MAI relative to the greatest mean annual increment (MAI) across years) from tree age 3 to 17 years for two hybrid poplar clones [*Populus deltoides* Bart. ex Marsh. × *P. nigra* L. ‘DN34′; *P. nigra* × *P. maximowiczii* A. Henry ‘NM6′] grown for phytoremediation at the former Rhinelander City Landfill in Rhinelander, WI, USA.

The clone × age interaction was significant for RELATIVE_MAI_ (*p* = 0.0305) (Table 1), with cubic equations exhibiting the best model fit for both clones (Figure 3A,B). Specifically, the coefficients of determination were *R*^2^ = 0.8865 for ‘DN34′ and *R*^2^ = 0.8572 for ‘NM6′. The growth curves for both clones represented three distinct developmental plantation stages. In particular, there was an early peak in growth rates through the first 4 years (i.e., relatively substantial increases in RELATIVE_MAI_). From five to 13 years, there was a steady increase in RELATIVE_MAI_, followed by a leveling off of RELATIVE_MAI_ (i.e., changes in growth rates < 5% annually) from years 14 to 17 (Figure 3A,B).

**Figure 4:**
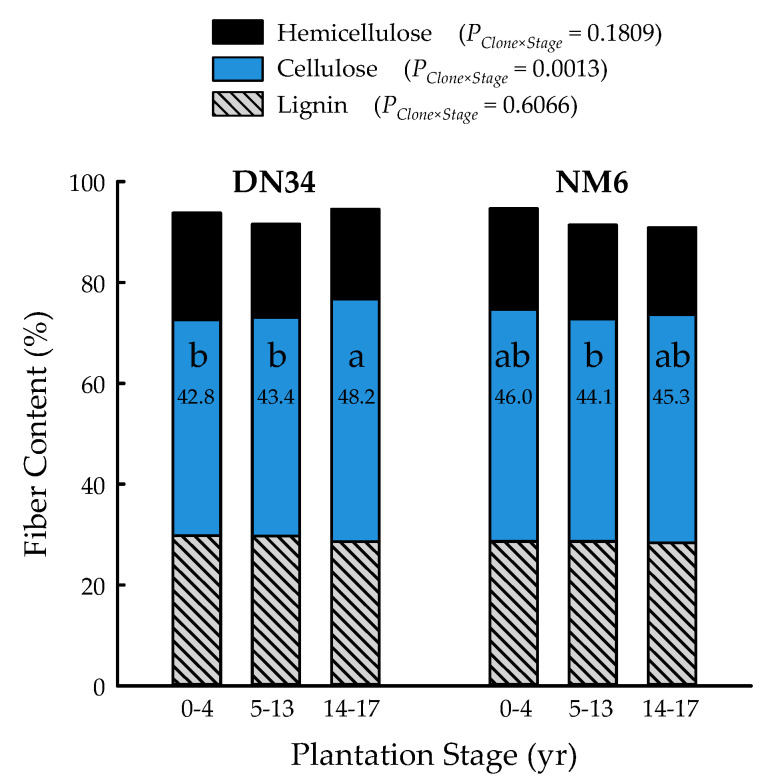
Percent hemicellulose, cellulose, and lignin of two hybrid poplar clones [*Populus deltoides* Bart. ex Marsh. × *P. nigra* L. ‘DN34′; *P. nigra* × *P. maximowiczii* A. Henry ‘NM6′] at three developmental plantation stages (0 to 4, 5 to 13, 14, to 17 years) grown for phytoremediation at the former Rhinelander City Landfill in Rhinelander, WI, USA. Cellulose values with the same letters are not different at *p* < 0.05.

The clone and plantation stage main effects were significant for lignin (*p* = 0.0468) and hemicellulose (*p* < 0.0001) fiber content, respectively, while the clone × plantation stage interaction governed cellulose content (*p* = 0.0013) (Table 1). Lignin was 3% greater for ‘DN34′ (29.4 ± 0.3%) than ‘NM6′ (28.6 ± 0.2%), across plantation stages. Hemicellulose content significantly decreased with tree age, with 20.6 ± 0.3%, 18.6 ± 0.2%, and 17.5 ± 0.3% for 0- to 4-, 5-, to 13-, and 14- to 17-year-old trees, respectively. Plantation stages 0 to 4 years and 14 to 17 years were significantly different than the overall mean of 18.9 ± 0.2% hemicellulose. Cellulose content ranged from 42.8 ± 0.7% (‘DN34′, 0 to 4 years) to 48.2 ± 0.9% (‘DN34′, 14 to 17 years), with an overall mean of 45.0 ± 0.4% (Figure 4). Trees of ‘DN34′ had the highest cellulose content during plantation stage 14 to 17 years, which was 12% and 11% greater than 0 to 4 and 5 to 13 years, respectively, which were not different from one another. For ‘NM6′, the cellulose content did not differ among plantation stages (Figure 4).

Differences between clones were negligible for height (*p* = 0.0588), total carbon (*p* = 0.5100), and branch moisture (*p* = 0.3334) (Table 2). The specific gravity of ‘DN34′ was 13% greater than ‘NM6′ (*p* = 0.0001), while ‘NM6′ outperformed ‘DN34′ for all other productivity- and wood-related traits. In particular, ‘NM6′ had 56% larger diameter, 236% greater leafless BIOMASS_MAI_, 233% greater total BIOMASS_MAI_, 229% greater leafless CARBON_MAI_, 243% greater total CARBON_MAI_, and 9% more bole moisture (*p* < 0.0001 for all traits) (Table 2).

Phytoaccumulation and phytoextraction in leaves, boles, and branches were clone- and tissue-specific (Table 3), with ‘DN34′ generally having higher uptake levels than ‘NM6′, and differences in elemental concentrations being present in leaves and branches at greater frequencies and magnitudes than in the boles. There were no differences in the uptake of total N, total C, Cd, Cr, Ni, or Pb between clones for any of the tree tissues (*p* > 0.05). In contrast, ‘DN34′ exhibited 41 to 310% significantly higher concentrations of Fe, Mn, and Na than ‘NM6′ in all three tissues (Table 3). The leaf, bole, and branch Fe concentrations were 117%, 212%, and 105% greater for ‘DN34′, respectively, while those for Mn were 63% (leaf), 310% (bole), and 94% (branch) higher. Sodium concentrations in ‘DN34′ leaves, boles, and branches were 41%, 155%, and 68% greater, respectively. For K, ‘DN34′ exhibited 22% higher phytoaccumulation into leaves and 16% greater uptake into branches; differences in the bole K concentrations were negligible (Table 3). Four tissue × element combinations had significantly greater concentrations for ‘DN34′ than ‘NM6′: (1) Branch_Mg_ +26%, (2) Branch_P_ +23%, (3) Leaf_Cl_ +30%, and (4) Bole_Zn_ +31%. In contrast to the general trend of ‘DN34′ having greater uptake than ‘NM6′, five tissue × element combinations had significantly greater concentrations for ‘NM6′ than ‘DN34′: (1) Leaf_Ca_ +85%, (2) Branch_Ca_ +34%, (3) Leaf_Al_ +53%, (4) Branch_Al_ +267%, and (5) Branch_Co_ +549%. There was one instance of ‘DN34′ and ‘NM6′ each having higher concentrations for the same element, depending on the tissue. In particular, ‘DN34′ had 139% more Cu than ‘NM6′ in branches, while ‘NM6′ exhibited 188% more Cu than ‘DN34′ in boles (Table 3).

### 2.2. Biofuels Recalcitrance

**Figure 5:**
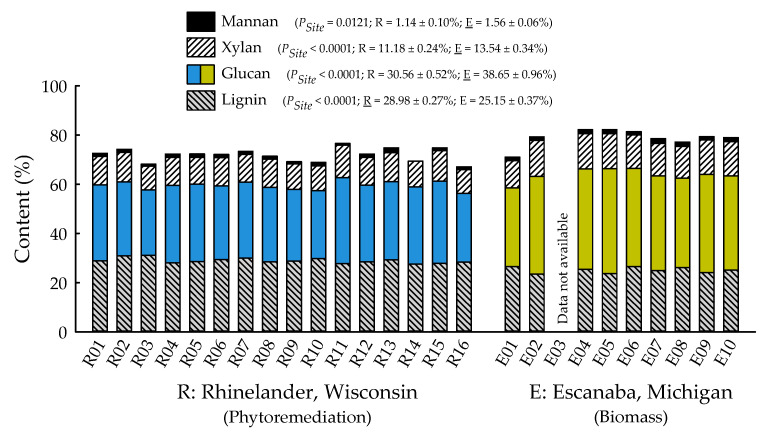
Percent mannan, xylan, glucan, and lignin across two hybrid poplar clones [*Populus deltoides* Bart. ex Marsh. × *P. nigra* L. ‘DN34′; *P. nigra* × *P. maximowiczii* A. Henry ‘NM6′] grown for phytoremediation at the former Rhinelander City Landfill in Rhinelander, WI, USA, and biomass production at the Michigan State University, Forest Biomass Innovation Center (FBIC) in Escanaba, MI, USA. Probability values for site main effects are listed in parentheses, along with means ± one standard error. R01 to R16 = trees 1 to 16 in Rhinelander; E01 to E10 = trees 1 to 10 in Escanaba.

**Figure 6:**
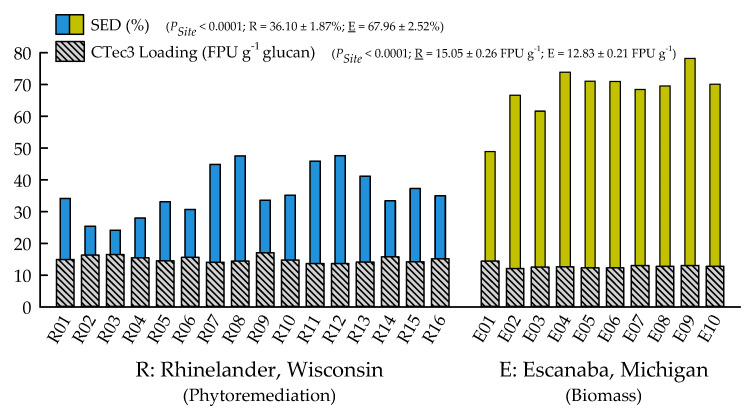
Substrate enzymatic digestibility (SED; %) and CTec3 loading (FPU g^−1^ glucan) across two hybrid poplar clones [*Populus deltoides* Bart. ex Marsh. × *P. nigra* L. ‘DN34′; *P. nigra* × *P. maximowiczii* A. Henry ‘NM6′] grown for phytoremediation at the former Rhinelander City Landfill in Rhinelander, WI, USA, and biomass production at the Michigan State University, Forest Biomass Innovation Center (FBIC) in Escanaba, MI, USA. The probability values for site main effects are listed in parentheses, along with means ± one standard error. R01 to R16 = trees 1 to 16 in Rhinelander; E01 to E10 = trees 1 to 10 in Escanaba.

**Figure 7:**
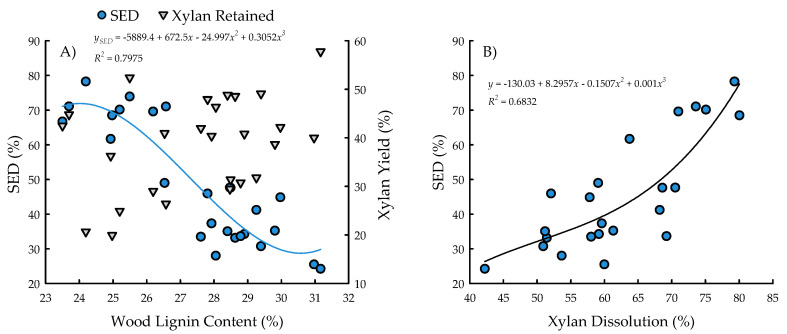
Substrate enzymatic digestibility (SED; %) and xylan yield (%) versus wood lignin content (%) (**A**) and SED versus xylan dissolution (%) (**B**) across two hybrid poplar clones [*Populus deltoides* Bart. ex Marsh. × *P. nigra* L. ‘DN34′; *P. nigra* × *P. maximowiczii* A. Henry ‘NM6′] grown for phytoremediation at the former Rhinelander City Landfill in Rhinelander, WI, USA. The relationship between xylan yield and lignin was not significant at *p* < 0.05.

**Figure 8:**
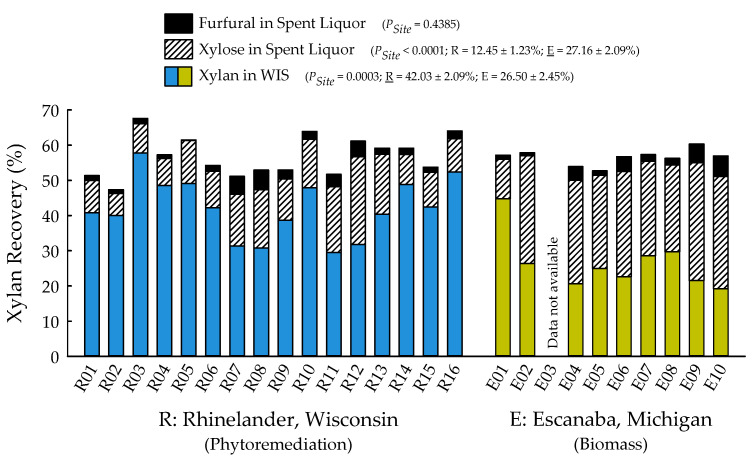
Percent recovery of furfural in spent liquor, xylose in spent liquor, and xylan in water insoluble solids (WIS) across two hybrid poplar clones [*Populus deltoides* Bart. ex Marsh. × *P. nigra* L. ‘DN34′; *P. nigra* × *P. maximowiczii* A. Henry ‘NM6′] grown for phytoremediation at the former Rhinelander City Landfill in Rhinelander, WI, USA, and biomass production at the Michigan State University, Forest Biomass Innovation Center (FBIC) in Escanaba, MI, USA. The probability values for site main effects are listed in parentheses, along with means ± one standard error. R01 to R16 = trees 1 to 16 in Rhinelander; E01 to E10 = trees 1 to 10 in Escanaba.

**Figure 9:**
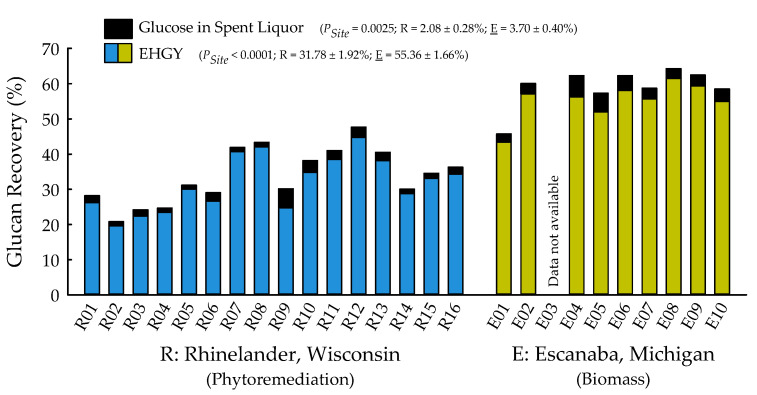
Percent glucose in spent liquor and enzymatic hydrolysis glucose yield (EHGY) across two hybrid poplar clones [*Populus deltoides* Bart. ex Marsh. × *P. nigra* L. ‘DN34′; *P. nigra* × *P. maximowiczii* A. Henry ‘NM6′] grown for phytoremediation at the former Rhinelander City Landfill in Rhinelander, WI, USA, and biomass production at the Michigan State University, Forest Biomass Innovation Center (FBIC) in Escanaba, MI, USA. Probability values for site main effects are listed in parentheses, along with means ± one standard error. R01 to R16 = trees 1 to 16 in Rhinelander; E01 to E10 = trees 1 to 10 in Escanaba.

With the exception of recovery of furfural in spent liquor (*p* = 0.4385), the site main effects were significant for all biofuels recalcitrance traits (*p* < 0.05), while clone main effects and site × clone interactions were negligible (Table 1). Mannan, xylan, and glucan content was 37%, 21%, and 26% greater for biomass trees growing in Escanaba, Michigan than phytoremediation trees growing in Rhinelander, Wisconsin. In contrast, Rhinelander phytoremediation trees exhibited 15% greater lignin content than Escanaba biomass trees (Figure 5). For substrate enzymatic digestibility (SED), Escanaba biomass trees were 88% higher than Rhinelander phytoremediation trees, even though the latter had 17% more CTec3 loading than their Escanaba counterparts (Figure 6). The relationship between SED and lignin content, as well as SED and xylan dissolution, were significant, with cubic equations exhibiting the best model fit and coefficients of determination of *R*^2^ = 0.7975 and *R*^2^ = 0.6832, respectively (Figure 7). However, these relationships were different. In particular, there was an inverse relationship between SED and lignin content, with SED decreasing as wood lignin content increased, while SED increased with increasing xylan dissolution. The relationship between xylan retained in water insoluble solids (WIS) and lignin content was not significant (Figure 7). Despite the aforementioned lack of significance of furfural recovery in spent liquor between sites, the recovery of xylose in spent liquor was 118% greater for Escanaba biomass trees than Rhinelander phytoremediation trees, and the xylan retained in WIS was 59% greater for Rhinelander than Escanaba (Figure 8). Both enzymatic hydrolysis glucose yield (EHGY) and glucose recovery in spent liquor were the highest for Escanaba, with biomass trees exhibiting 74% and 78% greater glucan recovery than Rhinelander phytoremediation trees, respectively (Figure 9).

## 3. Discussion

The long-term success of phytoremediation systems is, by definition, quantified and valued based on contaminant stabilization, filtration, degradation, extraction, and/or volatilization into less harmful constituents [43,44]. While rotation-age phytoaccumulation and phytoextraction of carbon, nitrogen, and inorganic pollutants into leaves, boles, and branches of poplar clones ‘DN34′ and ‘NM6′ was a major focus of the current study, equally important results were elucidated for ecosystem services, such as aboveground biomass productivity and carbon storage potential. Oftentimes, such ecosystem services are not evaluated given resource constraints that are associated with budgets, personnel, and/or project duration [7]. However, combining these services with phytoremediation potential is useful for developing stand-level silvicultural prescriptions that relate to landscape-level phytomanagement of ecosystem services [21,45]. In addition to long-term phytoremediation effectiveness and the provision of ecosystem services throughout poplar rotations, data are also lacking for the identification and post-processing of the wood for specific applications once the trees have reached their phytoremediation life span [5,36]. More often than not, end uses of the feedstocks are not identified during project planning nor implementation. To address this need to inform end-use decisions, biofuels recalcitrance traits were compared between Rhinelander phytoremediation trees and Escanaba biomass trees in order to test whether the feedstocks grown on contaminated soils exhibited similar biofuel yields as their traditionally-grown, agricultural counterparts. Overall, the clone effects were negligible and site-related factors governed bioconversion potential. Rhinelander phytoremediation trees exhibited 15% greater lignin content than Escanaba biomass trees, contributing to 46% lower glucose yield for phytoremediation-grown trees.

The results of the current study corroborated the general trend of ‘NM6′ having greater biomass productivity, carbon storage potential, and additional ecosystem services than ‘DN34′ in the North Central United States [20,46]. For example, ‘NM6′ had 28% greater height than ‘DN34′ in a phytoremediation system with soils heavily contaminated with petroleum hydrocarbons near Gary, IN, USA (41.5° N, 87.3° W) [47]. In contrast, 11-year-old ‘DN34′ and ‘NM6′ trees that were grown for phytoremediation of salts, metals, and nitrates were not significantly different, despite ‘NM6′ having 6% greater biomass and carbon [7,48]. While both clones perform as generalists throughout the region, ‘DN34′ has a much greater latitudinal range, with ‘NM6′ experiencing reduced growth and increased disease incidence (e.g., Septoria canker, *Septoria musiva* Peck) below 43° N latitude [17,18,49]. At the Rhinelander landfill in the current study, ‘NM6′ exhibited 3.4 times greater biomass productivity and carbon storage than ‘DN34′, yet both the clones had similar carbon isotope discrimination (Δ), which differed for tree age rather than genotype (Table 1; Figure 1). Water use efficiency (WUE) and δ^13^C stable isotope ratios (and, therefore, Δ) have shown mixed results for their relationships with plant productivity. Previous studies have reported both the existence [50,51,52] and lack [53,54,55] of correlations among WUE, δ^13^C, or ∆ with growth parameters. For the Rhinelander phytoremediation trees, BIOMASS_MAI_ was positively significantly correlated with Δ across clones and ages (*p* < 0.0001; *r* = 0.34) and within individual clones (‘DN34′ *p* < 0.0001, *r* = 0.49; ‘NM6′ *p* < 0.0001, *r* = 0.46) (data not reported). These positive correlations between biomass production and ∆ suggested that high productivity and low WUE characterized ‘DN34′ and ‘NM6′, which were likely driven by variations in stomatal conductance, resulting in high BIOMASS_MAI_ at the expense of water use [56]. Therefore, despite being beyond the scope of the current study, ∆-assisted, multi-trait selection of productive clones should be carefully considered in future efforts, focusing on both growth and high WUE [51,57]. Such efforts are important, as they take into account linkages between WUE and phytoremediation that are associated with potential stress impacts from landfill soil properties and contamination sources [10,31,32,33,34,35].

In the current study, phytoaccumulation and phytoextraction were clone- and tissue-specific, with ‘DN34′ generally exhibiting higher pollutant concentrations in leaves, boles, and branches. Assessed independently based solely on these concentrations, it could be assumed that ‘DN34′ was more efficient at remediating the landfill. However, ‘NM6′ outperformed ‘DN34′ for all biomass- and carbon-related traits (Table 2). While using a mass balance approach, calculated stand-level, mean annual elemental uptake (PHYTO_MAI_) across contaminants was 28% (Mn) to 657% (Cr) greater for ‘NM6′ (Table 4), indicating its phytoremediation superiority despite ‘DN34′ having generally higher pollutant concentrations in all tree tissues. The universal phytoaccumulation/phytoextraction advantage of ‘DN34′ for Fe, Mn, and Na was of particular note (Table 3). Despite ‘DN34′ exhibiting 41 to 310% significantly higher concentrations than ‘NM6′ in all three tissues, ‘NM6′ had 28%, 54%, and 60% greater PHYTO_MAI_, respectively, thus illustrating the need to integrate ecosystem services with phytoprocess-related information before determining overall phytoremediation effectiveness [21].

In contrast to ecosystem services and physiology, neither genotype nor genotype × environment interactions influenced biofuels recalcitrance traits. Instead, site effects governed substrate enzymatic digestibility (SED) and the recovery of most wood constituents (Figure 8). These results are unique given the broad genetic diversity within the genus *Populus* [58,59] and, more specifically, differences between these clones for various applications, ranging from biomass to phytotechnologies [7,18,28]. It is uncommon for clonal effects to be non-existent for such poplar clones, and even more uncommon for clonal responses to their environments to be negligible [17], although geographically robust clones have been identified [60,61]. Identifying clonal differences is a major component of successful pretreatment of woody biomass (including poplars) for biofuels production [38]. For example, tests of *Populus tremuloides* Michx. ‘native aspen collection’, *Populus deltoides* Bartr. ex Marsh × *Populus nigra* L. ‘NE222′ and ‘DN5′ (which both belong to the same genomic group as ‘DN34′ in the current study), and ‘NM6′ (as in the current study) identified stark contrasts among the genotypes for bioconversion potential [41]. In particular, following dilute acid (DA) pretreatment, the native aspen exhibited 29% greater total monomeric sugar yield and 143% greater ethanol yield than ‘NM6′, while sulfite pretreatment for overcoming recalcitrance of lignocelluloses (SPORL) pretreatment produced 12% and 82% greater sugar and ethanol yield for native aspen versus ‘NM6′, respectively. For both pretreatments, bioconversion potentials of ‘NE222′ and ‘DN5′ were between that of aspen and ‘NM6′ [41].

However, the results of the current study are unique, in that they elicited differences in site conditions (rather than genotypes) between the Rhinelander landfill trees and Escanaba biomass trees. Overall, climate conditions between the two sites were very similar throughout their respective rotations (Table 5), with the average growing season temperature differing by 0.3 °C and neither site experiencing extended periods of severe or extreme drought. However, water availability may have been a key growth-regulating factor contributing to recalcitrance between sites [52,55]. In particular, the maximum available soil water for the top 100 cm at Escanaba was nearly double that of Rhinelander (i.e., 15.01 versus 7.43 cm, respectively) (data from [62]), despite minimal differences in mean annual precipitation (i.e., 511 versus 571 mm, respectively) (Table 5). According to Monclus et al. [55], the selection of drought tolerant poplar genotypes may support movement of genetic material from moist sites to drier sites with less available water. Our assertion is that both ‘DN34′ and ‘NM6′ may be relatively drought tolerant (compared among the hundreds of genotypes tested in the North Central United States) and, therefore, clonal differences were negligible despite site-related variability in water availability. In addition, soil texture was similar (Rhinelander: sandy loam; Escanaba: fine sandy loam), yet the soil pH varied greatly, with Rhinelander being very strongly acidic (pH = 4.8) and Escanaba slightly alkaline (pH = 7.5). Despite this difference in edaphic conditions, ideal pH for poplars ranges from 5.0 to 7.5 [63], which indicates that Rhinelander and Escanaba are on opposite ends of the spectrum, yet still within its general limits. Typically, soils closer to pH of 4.0 have caused impacts to establishment and subsequent biomass production [64]. Variability in chemical soil properties may also have influenced recalcitrance levels between the clones, but chemical analytical testing of Escanaba soils (along with phytoaccumulation and phytoextraction) was beyond the scope of the current study. Such testing is warranted for future investigations.

Overall, from the standpoint of wood chemical composition, Rhinelander phytoremediation trees had a higher lignin content and lower cellulose content than their Escanaba biomass counterparts (Figure 5). Increased lignification has been associated with drought-stress resistance, and examples of increased lignin synthesis were found for almost all abiotic stresses [65]. For example, responses for seedlings of white elm (*Ulmus laevis* Pall.) that were subjected to arsenic (As) contamination underpinned that some forms of pollution caused increased lignin content [66]. Specifically, Waliszewska et al. [66] reported a 10% increase in lignin above and beyond the 23.75% lignin content in reference material. Similarly, for the genus *Populus*, increased lignin deposition was observed in cuttings of *Populus tremula* L. × *P. tremuloides* hybrids that were exposed for 14 days to a chilling temperature of 10 °C. Lignin content started to increase within two days of treatment and continued until the end of chilling exposure [67].

The lignin content of individual trees ranged from 25.6 to 32.3% in the current study, which corroborated previous results [68,69,70]. In a review of compositional traits of poplars used as biofuels feedstocks, Sannigrahi et al. [69] reported a range in wood lignin content across poplar species and hybrids of 21.5 to 27.2%, while Klašjna et al. [68] tested four-year-old trees of 40 eastern cottonwood (*Populus deltoides*) clones and reported 19.8 to 24.8% wood lignin content, with a mean of 22.2%. When discussing phytoremediation potential of bioenergy plants, the impact on lignin content is not a major focus [71]. However, it has been shown that genotypes and species with high lignin content exhibited properties that were appropriate for thermochemical conversion to biofuels, while those with low lignin content were more suitable for biochemical conversion [71]. The lignin contents of extractive-free poplar wood samples that were determined by the acetyl bromide method ranged from 23.4 to 32.1%, with associated calorific values measured with a combustion calorimeter ranging from 17,260 to 19,767 J g^−1^, revealing that intraspecific variations in lignin and energy contents were independent of one another and that lignin content was a poor predictor of energy content [70].

Biofuels recalcitrance results of the current study are in agreement with previous studies [41,72]. Work has been conducted for testing biofuels yields from ‘NE222′ grown for biomass production while using SPORL pretreatment [39] and acid hydrotropic fractionation [40,73] to evaluate substrate enzymatic digestibility (SED), sugar yield, production of furfural, and other related traits. The lower lignin content in the Escanaba biomass trees may have resulted from differences in the presence of tension wood, given that tension wood contains higher glucan content and undergoes higher enzymatic conversion to fermentable sugars [74]. In the current study, under the same pretreatment conditions, Escanaba trees had much better SED (i.e., were less recalcitrant) than Rhinelander trees (Figure 6), indicating some site-related factors in their biofuel potential (see above).

For enzymatic cellulose saccharification, the variation in cellulase loading was minimal, with most samples being dosed at approximately 10 FPU g^−1^ of substrate glucan for all pretreated water insoluble solids (WISs); this was due to the hydrolysis experiments being carried out before wood carbohydrate data were available, and instead using a target of 64% substrate glucan from previous poplar research [39,41]. The elevated cellulose content of the Escanaba trees, as well as their pretreated WISs, resulted in lower CTec3 loading (per g glucan base) than the loadings for Rhinelander trees. Similarly, despite their aforementioned lower cellulase loadings, the higher SED of Escanaba trees can be attributed to their lower lignin content that facilitated more efficient pretreatment, which resulted in greater hemicellulose (xylan) dissolution and greater digestible substrates. It has been recognized that xylan dissolution is critically important to improve SED [75]. In the current study, the amount of xylan retained in WISs increased with increasing lignin content, resulting in a decrease of SED as lignin increased (Figure 7). Furthermore, the evaluation of the mass balance of xylan recovery showed that a substantial amount of xylan remained in pretreated WISs (Figure 8), especially those from Rhinelander trees that had high lignin content. These results corroborated those of Zhang et al. [39]. In particular, a substantial amount of dissolved xylan was hydrolyzed into xylose, dehydration of the dissolved xylan to furfural was minimal, and the residual xylan was mostly comprised of xylo-oligomers that were not analyzed [39]. Overall, the results from the current study showed more xylan dissolution and more xylose from Escanaba trees than Rhinelander trees given the higher lignin content of the Rhinelander trees that protected them from xylan dissolution. Evaluation of the mass balance of cellulose recovery showed that amounts of cellulose (i.e., glucan) retained after SPORL pretreatment on washed WISs were approximately 75% or higher, while the amount of dissolved glucan as glucose was less than 6% in spent liquor (Figure 9). The balances were oligomeric glucose in the spent liquor. The evaluation of total glucose recoveries from dissolved glucose in spent liquors and enzymatic hydrolysis glucose yields (EHGY) showed that Escanaba biomass trees had an average total glucose yield of approximately 65% of the wood cellulose. In contrast, Rhinelander phytoremediation trees had a total glucose yield of approximately 35%, due to lower enzymatic cellulose conversion.

During cellulosic hydrolysis, both hemicellulose and lignin restrain sugar release, yet there have been positive impacts while using pretreatments to partly remove them [76], such as SPORL tested in the current study [39,41]. Among the *Salicaceae* family, willow (*Salix* spp.) species and hybrids have been extensively studied for biofuels, bioenergy, and bioproducts [77]. Ray et al. [78] tested numerous willow genotypes and noted that wood lignin content was not correlated with the accessibility of glucan to enzymatic saccharification. They stated that minimal evidence exists to support assumptions that high glucan and low lignin contents are preferred qualities for all biofuel feedstocks [78]. Their findings showed that high lignin content of untreated biomass is not necessarily detrimental to final glucose yields and, therefore, selecting for genotypes with high lignin content may be beneficial, given the possible energy rewards of residue combustion [78], which may be a potential end use of phytoremedation-grown trees.

In addition, when considering poplar biomass as a feedstock for biofuels, elemental composition, as well as carbohydrate and lignin content and composition, are all relevant traits [69]. Lignin is mostly comprised of syringyl units, rendering it more labile to chemical pretreatments used for biomass to biofuels conversion. Bose et al. [79] used an optimized nitrobenzene oxidation method to determine the S/G ratios of 13 poplar samples from two different sites and obtained S/G ratios that range from 1.0 to 1.7. Further, they reported an inverse linear relationship (*R*^2^ = 0.85) between decreasing lignin content and increasing S/G ratios [79]. Davison et al. [80] studied the effects of varying S/G ratios and lignin content of poplars on xylose release during dilute acid hydrolysis. They tested poplar clones with natural S/G variation (from 1.8 to 2.3) and differences in lignin content (22.7 to 25.8%) and reported that small decreases in the S/G ratio resulted in significant increases in xylose release after dilute sulphuric acid hydrolysis [80]. Studer et al. [72] tested undomesticated *Populus trichocarpa* trees and showed a strong negative correlation between sugar release and lignin content only for pretreated samples with an S/G ratio < 2.0. For S/G ratios > 2.0, sugar release was generally higher, and the negative influence of lignin was less pronounced. When examined independently, only glucose release was correlated with lignin content and S/G ratio in this manner, whereas xylose release depended on the S/G ratio alone [72]. Although clones were not significantly different in the current study, these previous results warrant future testing of a greater number of unrelated genotypes in phytoremediation versus biomass applications for biofuels conversion and related traits. Studying parameters that are related to ecosystem services, physiology, and biofuels recalcitrance are complementary for clonal selection and they could increase phytoremediation effectiveness at landfills and similar phytotechnology sites.

## 4. Materials and Methods

### 4.1. Clone and Site Selection

Two hybrid poplar clones that belong to different genomic groups were tested: *Populus deltoides* Bartr. ex Marsh × *P. nigra* L. ‘DN34′ and *P. nigra* × *P. maximowiczii* A. Henry ‘NM6′. These genotypes are the most widely-planted of all commercial and experimental clones in the region [17,18] and, therefore, were readily available for phytotechnologies when the sites of the current study were established.

The former Rhinelander City Landfill in Rhinelander, WI, USA (45.6° N, 89.4° W) was the phytoremediation study site [27], while the Forest Biomass Innovation Center (FBIC) of Michigan State University in Escanaba, MI (45.8° N, 87.2° W) was the biomass production study site [62]. Table 5 illustrates site- and climate-related information, including latitude/longitude, county, year planted, stocking, and tree age, height, and diameter at harvest. In addition, climate and drought data were averaged monthly across each growing season (April to October) and summed/averaged to determine annual values for fifteen years at Rhinelander (2001 to 2015, when the trees were three to 17 years old) and seven years at Escanaba (2003 to 2009, when the trees were three to nine years old). The National Oceanic and Atmospheric Administration (NOAA) National Climate Data Center (NCDC, https://www.ncdc.noaa.gov/cdo-web/) was accessed to obtain precipitation (P, mm), and average (T_avg_, °C), maximum (T_max_, °C), and minimum (T_min_, °C) air temperatures from Rhinelander (USW00004803) and Escanaba (USC00201802) weather stations. The difference between maximum and minimum temperatures (i.e., T_diff_ = T_max_ − T_min_, °C) was calculated for each site. The United States Drought Monitor (https://droughtmonitor.unl.edu/) was accessed in order to obtain drought index scores according to percent area within each county (i.e., Oneida, Wisconsin; Delta, Michigan) belonging to the following drought index categories: D0 (abnormally dry), D1 (moderate drought), D2 (severe drought), and D3 (extreme drought).

Given the phytoremediation objectives at Rhinelander, the physical and chemical soil properties for the landfill were tested and they are reported in Table 6. Phytoremediation was not studied at Escanaba and, therefore, the soil properties were not tested for the biomass planting. At the Rhinelander landfill, a 30- to 60-cm cap that consists of gravelly, mixed soil was installed in the late 1980s. In 1999, the trees were planted directly into this cap [27]. For the current study, one soil sample was collected 0.5 m from the base of each experimental tree (i.e., eight trees per clone, sixteen trees total) in the north cardinal direction (i.e., 0° N) while using a stainless steel soil core sampler (AMS Inc., American Falls, ID, USA) with a plastic liner measuring 3.8 cm diameter × 30 cm length (i.e., soils were sampled to a depth of 30 cm). The soil samples were brought back to the USDA Forest Service, Northern Research Station (USFS NRS) in Rhinelander and stored at 5 °C until processing, which consisted of carefully extracting soil from the liners at lengths (i.e., depths) of 0 to 10 cm and 10 to 30 cm. The soils were air-dried and separated into two portions, by depth. Samples of the first portion were sent to Waypoint Analytical (Memphis, TN, USA) for soil texture determination. According to the methods of Zalesny et al. [81], soils of the second portion were sieved in order to pass through a 2-mm mesh screen and then analyzed at the USFS NRS in Rhinelander for pH, chloride (Cl^−^), total nitrogen (N), total carbon (C), aluminum (Al), cadmium (Cd), calcium (Ca), cobalt (Co), chromium (Cr), copper (Cu), iron (Fe), potassium (K), magnesium (Mg), manganese (Mn), sodium (Na), nickel (Ni), lead (Pb), and zinc (Zn), as well as being sent to Northern Lake Service (Crandon, WI, USA) for analysis of phosphorus (P) (Table 6).

A Fisher Scientific Accumet^TM^ XL-50 m (Thermo Fisher Scientific, Carlsbad, CA, USA) was used to measure pH and Cl^−^. Specifically, pH was measured while using a Fisherbrand^TM^ AccuCap^TM^ capillary junction pH electrode (Thermo Fisher Scientific, Carlsbad, CA, USA). The meter was calibrated using buffer standards that were prepared from Fisher Buffer Salt of pH 4.01 and 7.41 (Thermo Fisher Scientific, Carlsbad, CA, USA). Chloride was measured using a Fisherbrand^TM^ Accumet^TM^ solid-state half-cell Cl^−^-specific electrode using standards prepared from a 1000 mg Cl^−^ L^−1^ standard (Ricca Chemical, Arlington, TX, USA).

The soil concentrations of total N and total C were analyzed using a Flash EA1112 N-C analyzer (Thermo Electron, via CE Elantech, Inc., Lakewood, NJ, USA) with a model MAS 200 autosampler, while Al, Cd, Ca, Co, Cr, Cu, Fe, K, Mg, Mn, Na, Ni, Pb, and Zn were measured using an Agilent AA240FS fast sequential atomic absorption spectrometer (using Mehlich-3 extracting solution) (Agilent Technologies, Santa Clara, CA, USA). Phosphorus was determined while using an axial aligned Varian 720-ES ICP Optical Emission Spectrometer with a CCD detector (Varian Inc., Palo Alto, CA, USA).

### 4.2. Field Sampling

At Rhinelander, the diameter at breast height (i.e., DBH at 1.37 m) was measured to the nearest 0.1 cm, DBH was marked on eight randomly-selected trees per clone, and the sixteen study trees were felled. On the ground, the total tree height was measured to the nearest 0.1 m, and aboveground fresh biomass of leaves, boles, and branches was determined to the nearest 0.1 kg. Branch subsamples were collected for each tree and weighed to the nearest 0.1 kg for fresh biomass determination. At DBH, cross-sectional disks were harvested and weighed to the nearest 0.1 g for fresh mass determination. A 61-cm length of bole wood was extracted from directly above the DBH disk and chipped. Branch subsamples, disks, and chips were transported to the analytical laboratory at the USFS NRS in Rhinelander and then oven-dried at 55 °C until constant mass at a precision of 0.1 kg. Moisture content of bole and branch wood was calculated, and aboveground dry biomass per tree was determined to the nearest 0.1 kg. The mean annual increment (MAI) of harvest biomass (BIOMASS_MAI_) was calculated by dividing aboveground dry biomass [both leafless (boles + branches) and total biomass (leaves + boles + branches)] by harvest age (i.e., 17 years). After the dry biomass determinations were completed, a subsample of chips was ground and sent to the USFS Forest Products Laboratory (USFS FPL) in Madison, Wisconsin, for biofuels recalcitrance testing, which is described below. The leaves were ground, and all leaf and wood ground samples were analyzed in order to determine leaf, bole, and branch concentrations of total N, total C, Al, Cd, Ca, Cl (just leaves), Co, Cr, Cu, Fe, K, Mg, Mn, Na, Ni, P, Pb, and Zn, with methods being the same as for soil analyses.

At Escanaba, four trees per clone were harvested nine years after planting for a companion study [62] while using the same procedure as for the Rhinelander phytoremediation trees. An additional 11 and 15 trees of ‘DN34′ and ‘NM6′, respectively, were harvested for on-site FBIC needs. As with Rhinelander trees, a 61-cm length of bole wood was extracted above DBH and then chipped for all 34 trees. A subsample of wood chips from each Headlee et al. [62] tree was collected, as well as a bulked subsample of wood chips, by clone, from the FBIC trees. The ten wood chip subsamples from the Escanaba biomass trees were sent to the USFS FPL for biofuels recalcitrance testing.

### 4.3. Ring Width Measurements and Laboratory Sampling

One cross-sectional area of each Rhinelander DBH disk was sanded, wetted, and imaged for companion studies. The sanded disks were cut in half along a plane extending through the pith, and the cut face of each half-disk was sanded. One half-disk was used for specific gravity determination while using the same methods as Headlee et al. [62]. From the remaining half-disk, a wafer that was free of bark and defects was harvested. From each wafer, a Wild E5-86550 Stereomicroscope with 10× power eyepieces and a 6× power setting of the objective (Wild Company, Heerbrugg, Switzerland) was used in order to define and measure annual ring width measurements and total growth ring measurements [i.e., inside bark diameter (DIB)] to the nearest 0.1 cm. Following ring measurements, wood samples that ranged from 2 to 10 mg dry mass were extracted from each annual ring for the total N and total C analyses described above, and another set with samples ranging from 1 to 2 mg dry mass were extracted from each annual ring for carbon isotope (δ^13^C) analyses.

### 4.4. Total Carbon and Wood Composition

Total carbon of annual rings was determined while using a Flash EA1112 N-C analyzer (Thermo Electron, via CE Elantech, Inc., Lakewood, NJ, USA) with a model MAS 200 autosampler. The carbon concentrations were combined with BIOMASS_MAI_ values to generate CARBON_MAI_ estimates at harvest (both leafless (boles + branches) and total (leaves + boles + branches)).

Ground wood samples were combined by plantation stage (i.e., 0 to 4, 5 to 13, and 14 to 17 years) for each Rhinelander tree. In order to determine hemicellulose, cellulose, and lignin composition, neutral detergent fiber (NDF), acid detergent fiber (ADF), and acid detergent lignin (ADL) were measured with an ANKOM 200 Fiber Analyzer (ANKOM Technology, Macedon, NY, USA), according to methods that were outlined by ANKOM [82]. Hemicellulose content was calculated by subtracting ADF from NDF (i.e., NDF-ADF), cellulose by subtracting ADL from ADF (i.e., ADF-ADL), and lignin as ADL (plus ash). A modification to ANKOM’s protocol was used, drying samples at each stage for 16 h rather than the standard 2 to 4 h. As a result of this extended drying time (and the potential for an increase of pore size and loss of lignin from the ANKOM F57 filter bags), the cellulose values were greater than expected and lignin values less than expected for poplars [69]. We developed and applied a correction factor to the cellulose and lignin data generated from the ANKOM analyses based on the values that were obtained from the USFS FPL during biofuels recalcitrance testing for these identical trees. These corrected data were used in all analyses.

### 4.5. Carbon Isotope (δ^13^C) Analyses and Discrimination (Δ) Calculations

A Finnigan^TM^ MAT DELTA^plus^XL Mass Spectrometer (Thermo Fisher Scientific, Waltham, MA, USA) was operated in continuous flow mode and connected to a Costech 4010 Elemental Combustion System Elemental Analyzer (Costech Analytical Technologies, Inc., Valencia, CA, USA) to measure stable carbon isotope (δ^13^C) values. Caffeine (IAEA-600), cellulose (IAEA-CH-3), and Acetanilide (laboratory standard) reference standards were analyzed every ten samples for isotopic corrections and assigning the data to the appropriate isotopic scale. The data were corrected while using regression analyses, with combined uncertainty (analytical uncertainty and average correction factor) for δ^13^C of ± 0.03 to 0.15‰ (VPDB).

Carbon isotope discrimination (Δ) was calculated according to Farquhar et al. [30]:

(1) $$\Delta \;=\;\frac{\delta_{a}-\delta_{p}}{1+\delta_{p}}$$

where δ_a_ equals the isotopic composition of air that is assumed to be −8 ‰, and δ_p_ equals the isotopic composition (δ^13^C) of the wood sample analyzed. These Δ values were used in all analyses.

### 4.6. Annual Biomass Estimates

The following model from Bond–Lamberty et al. [83] was applied in order to calculate annual outside bark diameter (DOB) based on DIB from the Rhinelander trees:

DOB = DIB^1.27^ (2)

Genotype-specific biomass equations from Headlee and Zalesny [84] were used to calculate individual-tree biomass:

BIOMASS_DN34_ = 10^−1.27^ × DOB^2.55^ (3)

BIOMASS_NM6_ = 10^−0.63^ × DOB^2.00^ (4)

Individual-tree biomass estimates were multiplied by the stocking of 834 trees ha^−1^ in order to obtain stand-level biomass estimates, which were then divided by tree age to generate BIOMASS_MAI_ values for three to 17 years of tree growth. Annual RELATIVE_MAI_ values were determined based on the ratio of current-year BIOMASS_MAI_ to maximum BIOMASS_MAI_ for each of the 15 years of the study (i.e., for three- to 17-year-old trees).

### 4.7. Sulfite Pretreatment to Overcome Recalcitrance of Lignocelluloses (SPORL) Pretreatment

Rhinelander phytoremediation samples were received in ground form while the Escanaba biomass samples were received in chip form. Individual samples were oven dried on aluminum foil for a minimum of 12 h at 105 °C to determine the solids content for calculating chemical loading in the SPORL pretreatment. These oven-dried samples were then Wiley-milled (Thomas Scientific, Swedesboro, NJ, USA) to 30 mesh for compositional analyses. For comparison purposes between the particle sizes, one Escanaba sample (i.e., ES-09) was ground by placing 30 g into a 2-L Waring blender (Conair Corporation, Torrington, CT, USA) and running on high for one minute. This was repeated five times to accumulate enough material for pretreatment.

The SPORL pretreatment method was conducted for all of the samples using a dilute sulfite liquor and 100 g wood dry mass. The fresh sulfite liquor had a pH of 2.44. Chemical loadings on percent wood dry mass were 3% sodium bisulfite and 1.1% sulfuric acid (both ACS reagent grade; Sigma–Aldrich, St. Louis, MO, USA). The pretreatments were conducted using a liquor to wood ratio of 3:1 (v:w) in 1-L sealed cylindrical reactors, as described previously [39]. After loading, the reactors were mounted into a 21-L rotating wood pulping digester (1 rpm) that was heated by direct steam injection. The digester holds three reactors at a time. This tumbling was conducted for five minutes prior to the addition of steam in order to provide some mixing prior to the start of the reaction. After five minutes, the steam was injected into the interior of the digester with continuous removal of the condensate. In all runs, it took four minutes to reach the target temperature of 160 °C (note: this was the digester internal temperature and it was assumed that the reactors were in equilibrium). The digester was then held for 35 min. at 160 °C via manual valve control. At the end of the reaction time, the digester was relieved of pressure and quickly opened, after which the reactors were removed and cooled in a bucket of cold tap water. The elapsed time from the end of the reaction to cooled reactors was 14 min.

There were 33 samples in total, including the replicates, so a total of eleven digester runs were conducted over six different days. In order to account for potential time variations over the several days during which the reactions were conducted, each digester run included two Rhinelander phytoremediation samples and one Escanaba biomass sample.

After the reactors were cooled, they were opened and their contents poured into pre-weighed 1-L polyethylene bottles. An additional 150 g of DI water was used for each sample to thoroughly rinse the reactor and was combined with the contents in each bottle. The bottles were then weighed as a check of total mass recovery efficiency, which ranged from 96.5 to 98.9%.

Next, the solids and free liquid were separated using a 300 mesh nylon cloth. The free liquid (i.e., spent liquor) was transferred into a pre-weighed polyethylene bottle while the solids remained with the original bottle. Mass was recorded for each stream. An aliquot of solids and spent liquor was collected for total and dissolved solids measurements, respectively, along with pH and high performance liquid chromatography (HPLC) analysis of dissolved constituents while using an UltiMate 3000 HPLC System (Thermo Fisher Scientific, Carlsbad, CA, USA). The recovered solids were refined in an atmospheric refiner with D2B-505 plates at a gap of 0.25 mm while using 30 L of dilution water as washing. The resulting slurry was then dewatered in a canvas bag and vacuum pressed to a water insoluble solids (WIS) level of 20 to 30%. The dewatered solids were measured for solids content and subsamples were dried and ground with a Wiley mill (Thomas Scientific, Swedesboro, NJ, USA) for compositional analysis.

### 4.8. Enzymatic Hydrolysis

The refined WIS samples were enzymatically hydrolyzed on a shaker incubator at 50 °C. The solids loading was 2% in a 5.5-pH sodium acetate buffer (ACS reagent grade; Sigma–Aldrich, St. Louis, MO, USA). A commercial complex cellulase Cellic^®^CTec3 (i.e., CTec3) was complimentarily provided by Novozymes North America (Franklinton, NC, USA). CTec3 cellulase application was estimated at 10 FPU g^−1^ glucan. The actual cellulase loading per g glucan was calculated for each sample after subsequent carbohydrate analyses of the solids were completed.

A 2-g dry mass aliquot of each pretreated wood sample, based on the measured solids level, was added to a 125 mL Erlenmeyer flask. An appropriate volume of sodium acetate buffer was added to the flask after deduction for the volume of the 10:1 diluted CTec3 (217 FU mL^−1^) of 21.7 FPU mL^−1^ and water contained in the sample (ACS reagent grade; Sigma–Aldrich, St. Louis, MO, USA). The pH of the slurry was adjusted to 5.5 with calcium oxide powder. After the pH adjustment, 0.59 mL of 10:1 diluted CTec3 was added to each flask, which was covered with foil and placed on the shaker incubator. Approximately 1 mL samples were taken at 1, 2, 4, 6, 24, 48, and 72 h while using a pipette and placed into a 1.5 mL snap-top plastic vials.

The hydrolysis samples were centrifuged for three minutes in a small centrifuge to settle any insoluble solids. The samples were then measured using a YSI 2700 Biochemistry Analyzer (YSI Life Sciences, Yellow Springs, OH, USA). Replicate enzymatic hydrolyses were conducted on four Rhinelander samples and three Escanaba samples.

### 4.9. Chemical Composition Analyses

The washed WIS along with untreated poplar wood samples were Wiley-milled to 30 mesh for chemical composition analysis at the Analytical Chemistry and Microscopy Laboratory of the USFS FPL. The traditional two-step sulfuric acid analysis was first applied to the samples in order to solubilize carbohydrates. Hydrolysates were analyzed by high performance anion exchange chromatography with pulsed amperometric detection using a Dionex^TM^ ICS-5000 Capillary HPIC^TM^ System (Dionex Corporation, Sunnyvale, CA, USA), as described previously [85]. Klason lignin (acid insoluble) was quantified gravimetrically [86]. The spent liquors were analyzed by HPLC using an UltiMate 3000 HPLC System (Thermo Fisher Scientific, Carlsbad, CA, USA) for glucose, xylose, galactose, arabinose, mannose, formic acid, acetic acid, levulinic acid, hydroxymethylfurfural (HMF), and furfural, as described previously [87].

### 4.10. Experimental Design and Data Analysis

Height, diameter, BIOMASS_MAI_, CARBON_MAI_, specific gravity, bole and branch moisture, total N, total C, and all elemental concentration (Al, Cd, Ca, Cl, Co, Cr, Cu, Fe, K, Mg, Mn, Na, Ni, P, Pb, Zn) data were subjected to analyses of variance (ANOVA) and analyses of means (ANOM) while using SAS^®^ (PROC GLM; PROC ANOM; SAS INSTITUTE, INC., Cary, NC, USA) assuming a one-factor design comparing clones (i.e., ‘DN34′ versus ‘NM6′). In addition, ANOVA and ANOM analyses in SAS^®^ were used to test for differences using three separate two-way factorial designs: 1) two clones, fifteen tree ages (i.e., years 3 to 17), and their interactions for total C, Δ, BIOMASS_MAI_, and RELATIVE_MAI_; 2) two clones, three plantation stages (i.e., 0 to 4, 5 to 13, and 14 to 17 years), and their interactions for hemicellulose, cellulose, and lignin content; and, 3) two sites (i.e., Rhinelander, Escanaba), two clones, and their interactions for mannan, xylan, glucan, lignin, substrate enzymatic digestibility (SED), CTec3 loading, furfural, xylose, and glucose in spent liquor, xylan in water insoluble solids (WIS), and enzymatic hydrolysis glucose yield (EHGY). Fisher’s protected least significant difference (LSD) was used to identify significant differences among means at *p* < 0.05.

Regression analyses were conducted in SAS^®^ (PROC REG SAS INSTITUTE, INC., Cary, NC, USA) in order to develop predictive response curves for interactions between RELATIVE_MAI_ and tree age for both clones, as well as separate curves between SED and wood lignin content, and SED and xylan dissolution. The best-fit models were selected based on the highest coefficients of determination (i.e., *R*^2^).

## Figures and Tables

**Table 1:** Probability values from analyses of variance for: (1) two hybrid poplar clones [*Populus deltoides* Bart. ex Marsh. × *P. nigra* L. ‘DN34′; *P. nigra* × *P. maximowiczii* A. Henry ‘NM6′] grown for seventeen years at the former Rhinelander City Landfill in Rhinelander, WI, USA (annual tree ages are from three to seventeen years) (*clone × age*); (2) clones ‘DN34′ and ‘NM6′ at three developmental plantation stages (0 to 4, 5 to 13, 14, to 17 years) at the former Rhinelander City Landfill (*clone × stage*); (3) clones ‘DN34′ and ‘NM6′ grown for phytoremediation at the former Rhinelander City Landfill and biomass production at the Michigan State University, Forest Biomass Innovation Center (FBIC) in Escanaba, MI, USA (*site × clone*). Significant values are bolded.

	Clone	Age	Clone × Age
Total carbon (%)	0.3401	**0.0023**	0.4764
Carbon isotope discrimination (Δ, ‰)	0.9986	**<0.0001**	0.1440
BIOMASS_MAI_ (Mg ha^−1^ yr^−1^)	**<0.0001**	**<0.0001**	**<0.0001**
RELATIVE_MAI_	0.6116	**<0.0001**	**0.0305**
	**Clone**	**Stage**	**Clone × Stage**
Hemicellulose (%)	0.0995	**<0.0001**	0.1809
Cellulose (%)	0.5900	**0.0010**	**0.0013**
Lignin (%)	**0.0468**	0.2193	0.6066
	**Site**	**Clone**	**Site × Clone**
Mannan (%)	**0.0121**	0.8283	0.1919
Xylan (%)	**<0.0001**	0.9529	0.9341
Glucan (%)	**<0.0001**	0.4084	0.8779
Lignin (%)	**<0.0001**	0.5497	0.1504
Substrate enzymatic digestibility (SED) (%)	**<0.0001**	0.1422	0.8719
CTec3 loading (FPU g^−1^ glucan)	**<0.0001**	0.3324	0.8638
Furfural in spent liquor (%)	0.4385	0.7268	0.9538
Xylose in spent liquor (%)	**<0.0001**	0.2598	0.7119
Xylan in water insoluble solids (WIS) (%)	**0.0003**	0.5373	0.7516
Enzymatic hydrolysis glucose yield (EHGY) (%)	**<0.0001**	0.1619	0.5599
Glucose in spent liquor (%)	**0.0025**	0.1899	0.3148

MAI: mean annual increment; CTec3: commercial complex cellulase Cellic^®^CTec3 (Novozymes North America, Franklinton, North Carolina).

**Table 2:** Productivity- and wood-related traits (mean ± standard error) of two hybrid poplar clones [*Populus deltoides* Bart. ex Marsh. × *P. nigra* L. ‘DN34′; *P. nigra* × *P. maximowiczii* A. Henry ‘NM6′] grown for phytoremediation for seventeen years at the former Rhinelander City Landfill in Rhinelander, WI, USA. The probability values from analyses of variance are shown, with significant values bolded.

	DN34	NM6	*p*-Value
Height (m)	14.8 ± 0.5	16.7 ± 0.7	0.0588
Diameter (cm)	12.0 ± 0.4	18.7 ± 0.4	**<0.0001**
BIOMASS_MAI_ (Mg ha^−1^ yr^−1^), leafless	1.4 ± 0.1	4.7 ± 0.4	**<0.0001**
BIOMASS_MAI_ (Mg ha^−1^ yr^−1^), total	1.5 ± 0.1	5.0 ± 0.4	**<0.0001**
Total carbon (%)	48.3 ± 0.2	48.1 ± 0.2	0.5100
CARBON_MAI_ (Mg ha^−1^ yr^−1^), leafless	0.7 ± 0.1	2.3 ± 0.2	**<0.0001**
CARBON_MAI_ (Mg ha^−1^ yr^−1^), total	0.7 ± 0.1	2.4 ± 0.2	**<0.0001**
Specific gravity	0.363 ± 0.003	0.320 ± 0.007	**0.0001**
Bole moisture (%)	54.0 ± 0.3	59.0 ± 0.8	**<0.0001**
Branch moisture (%)	53.8 ± 0.9	52.7 ± 0.6	0.3334

MAI: mean annual increment.

**Table 3:** Elemental phytoaccumulation and phytoextraction (mean ± standard error) in leaves, boles, and branches of two hybrid poplar clones [*Populus deltoides* Bart. ex Marsh. × *P. nigra* L. ‘DN34′; *P. nigra* × *P. maximowiczii* A. Henry ‘NM6′] grown for seventeen years for phytoremediation at the former Rhinelander City Landfill in Rhinelander, WI, USA. Significant probability values from analyses of variance are bolded. Units are: % for N, C; g kg^−1^ for Ca, K, Mg, P; mg kg^−1^ for all other elements.

	Leaf			Bole			Branch		
	DN34	NM6	*p*-Value	DN34	NM6	*p*-Value	DN34	NM6	*p*-Value
Total nitrogen (N)	2.62 ± 0.12	2.33 ± 0.10	0.0777	0.28 ± 0.01	0.26 ± 0.02	0.3778	0.65 ± 0.04	0.56 ± 0.02	0.0510
Total carbon (C)	47.43 ± 0.10	47.23 ± 0.26	0.4819	48.27 ± 0.16	48.12 ± 0.15	0.5100	50.05 ± 0.15	49.84 ± 0.09	0.2465
Calcium (Ca)	10.05 ± 0.25	18.59 ± 1.62	**0.0001**	2.41 ± 0.43	1.84 ± 0.16	0.2390	3.39 ± 0.17	4.55 ± 0.36	**0.0116**
Potassium (K)	13.21 ± 0.82	10.80 ± 0.76	**0.0487**	5.39 ± 0.73	7.77 ± 1.03	0.0804	4.99 ± 0.25	4.29 ± 0.15	**0.0306**
Magnesium (Mg)	4.15 ± 0.24	4.25 ± 0.31	0.7938	1.13 ± 0.12	0.92 ± 0.04	0.1130	1.40 ± 0.08	1.11 ± 0.06	**0.0100**
Phosphorus (P)	2.07 ± 0.05	2.05 ± 0.08	0.7630	0.17 ± 0.01	0.14 ± 0.01	0.0646	0.86 ± 0.06	0.70 ± 0.03	**0.0360**
Aluminum (Al)	205.12 ± 31.06	314.15 ± 31.07	**0.0264**	245.40 ± 67.75	325.15 ± 36.67	0.3181	94.69 ± 21.66	347.98 ± 29.86	**<0.0001**
Cadmium (Cd)	0.12 ± 0.08	0.20 ± 0.13	0.6453	0.16 ± 0.13	0.02 ± 0.02	0.3006	0.12 ± 0.09	0.37 ± 0.09	0.0666
Chloride (Cl)	341.61 ± 25.09	262.58 ± 15.89	**0.0186**	Not tested	Not tested	-	Not tested	Not tested	-
Cobalt (Co)	6.37 ± 2.21	5.35 ± 1.21	0.6909	13.99 ± 2.18	12.21 ± 0.91	0.4624	0.55 ± 0.55	3.57 ± 1.29	**0.0493**
Chromium (Cr)	5.55 ± 0.83	3.20 ± 0.96	0.0860	1.03 ± 0.74	0.00 ± 0.00	0.1873	1.24 ± 0.63	5.12 ± 1.69	0.0505
Copper (Cu)	5.40 ± 0.81	8.37 ± 1.51	0.1044	2.81 ± 0.39	8.08 ± 0.97	**0.0002**	4.86 ± 0.53	2.03 ± 0.42	**0.0009**
Iron (Fe)	504.40 ± 39.27	232.02 ± 20.15	**<0.0001**	5.58 ± 1.45	1.79 ± 0.94	**0.0454**	89.12 ± 14.89	43.45 ± 6.65	**0.0141**
Manganese (Mn)	376.22 ± 27.99	231.50 ± 21.98	**0.0012**	65.11 ± 9.05	15.89 ± 4.06	**0.0002**	65.08 ± 8.61	33.56 ± 6.15	**0.0099**
Sodium (Na)	9.15 ± 0.66	6.49 ± 0.58	**0.0089**	14.62 ± 2.47	5.74 ± 1.70	**0.0103**	6.96 ± 0.62	4.14 ± 0.23	**0.0008**
Nickel (Ni)	4.32 ± 1.26	9.33 ± 2.33	0.0796	2.99 ± 0.86	4.34 ± 0.62	0.2277	4.63 ± 1.58	1.02 ± 0.89	0.0669
Lead (Pb)	7.00 ± 1.29	10.13 ± 1.64	0.1570	6.03 ± 2.02	9.69 ± 1.74	0.1921	5.26 ± 1.86	5.79 ± 1.83	0.8412
Zinc (Zn)	139.86 ± 12.08	169.37 ± 17.40	0.1854	25.63 ± 2.28	19.53 ± 1.28	**0.0348**	41.06 ± 2.09	43.45 ± 2.81	0.5055

**Table 4:** Stand-level, mean annual elemental phytoaccumulation and phytoextraction (PHYTO_MAI_) in aboveground dry leafless biomass of two hybrid poplar clones [*Populus deltoides* Bart. ex Marsh. × *P. nigra* L. ‘DN34′; *P. nigra* × *P. maximowiczii* A. Henry ‘NM6′] grown for seventeen years for phytoremediation at the former Rhinelander City Landfill in Rhinelander, WI, USA. Calculations were made from the average of bole and branch uptake values in Table 3 and BIOMASS_MAI_ (leafless) values in Table 2. Chloride (Cl) values are from leaf phytoaccumulation.

	PHYTO_MAI_			
	DN34	NM6	Difference	NM6 Advantage (%)
	------------ kg ha^−1^ yr^−1^ ------------			
Total nitrogen (N)	6.5	19.3	12.8	196
Total carbon (C)	688.2	2302.1	1613.8	234
Calcium (Ca)	4.1	15.0	11.0	270
Potassium (K)	7.3	28.3	21.1	290
Magnesium (Mg)	1.8	4.8	3.0	169
Phosphorus (P)	0.7	2.0	1.3	174
	------------- g ha^−1^ yr^−1^ ------------			
Aluminum (Al)	238.1	1581.9	1343.8	564
Cadmium (Cd)	0.2	0.9	0.7	368
Cobalt (Co)	10.2	37.1	26.9	264
Chromium (Cr)	1.6	12.0	10.4	657
Copper (Cu)	5.4	23.8	18.4	343
Iron (Fe)	66.3	106.3	40.0	60
Manganese (Mn)	91.1	116.2	25.1	28
Sodium (Na)	15.1	23.2	8.1	54
Nickel (Ni)	5.3	12.6	7.3	136
Lead (Pb)	7.9	36.4	28.5	360
Zinc (Zn)	46.7	148.0	101.3	217
Chloride (Cl) (Leaves)	34.2	78.8	44.6	131

**Table 5:** Site- and climate-related characteristics of the former Rhinelander City Landfill in Rhinelander, WI, USA, and the Michigan State University, Forest Biomass Innovation Center (FBIC) in Escanaba, MI, USA, where ecosystem services, physiology, and biofuels recalcitrance of poplars grown for landfill phytoremediation were compared to those grown for biomass.

Site	Rhinelander, WI	Escanaba, MI
Application	Phytoremediation	Biomass
Latitude, Longitude	45.6266° N, 89.3899° W	45.7708° N, 87.1978° W
County	Oneida	Delta
Year Planted	1999	2001
Stocking (trees ha^−1^)	834	1075
Tree Age at Harvest (yr)	17	9
Height at Harvest (m) (mean ± one standard error)	15.8 ± 0.5	12.0 ± 0.2
Diameter at Harvest (cm) (mean ± one Standard error)	15.4 ± 0.9	15.3 ± 0.7
Annual Precipitation (P) (mm)	571 ± 33	511 ± 11
Average Temperature (T_avg_) (°C)	13.2 ± 0.2	13.5 ± 0.1
Maximum Temperature (T_max_) (°C)	19.6 ± 0.3	20.0 ± 0.1
Minimum Temperature (T_min_) (°C)	6.7 ± 0.2	6.9 ± 0.1
Maximum—minimum Temperature (T_diff_) (°C)	12.9 ± 0.2	13.1 ± 0.0
Drought Index (abnormally dry) (D0) (%)	45.0 ± 8.5	53.5 ± 4.8
Drought Index (moderate drought) (D1) (%)	25.1 ± 8.3	22.1 ± 3.2
Drought Index (severe drought) (D2) (%)	8.5 ± 3.9	9.3 ± 2.0
Drought Index (extreme drought) (D3) (%)	0.8 ± 0.8	0.2 ± 0.1

Climate and drought data values are means ± one standard error across each growing season (April to October) of the fifteen years reported in the study when the phytoremediation trees were 3 to 17 years old in Rhinelander, WI, USA, and the seven years when the biomass trees were 3 to 9 years old in Escanaba, MI, USA. Climate data source: National Oceanic and Atmospheric Administration (NOAA) National Climate Data Center (https://www.ncdc.noaa.gov/cdo-web/). Drought index source: United States Drought Monitor (https://droughtmonitor.unl.edu/); percent of area within the county in each category (D0 to D3).

**Table 6:** Physical and chemical soil properties of the former Rhinelander City Landfill in Rhinelander, WI, USA, where ecosystem services, physiology, and biofuels recalcitrance of poplars grown for landfill phytoremediation were tested.

	Soil Depth (cm)	
	0 to 10	10 to 30
Texture	Sandy loam	Sandy loam
Sand (%)	61.9	69.5
Silt (%)	35.2	26.1
Clay (%)	2.9	4.4
pH	4.79 ± 0.06	4.51 ± 0.03
Total nitrogen (%)	0.18 ± 0.01	0.12 ± 0.01
Total carbon (%)	2.41 ± 0.12	1.31 ± 0.08
	--------------------- mg kg^−1^ ---------------------	
Aluminum (Al)	821.55 ± 28.08	1137.89 ± 25.43
Calcium (Ca)	667.23 ± 42.17	336.00 ± 24.39
Cadmium (Cd)	0.03 ± 0.01	0.05 ± 0.01
Chloride (Cl)	0.74 ± 0.09	0.90 ± 0.07
Cobalt (Co)	0.72 ± 0.06	0.87 ± 0.06
Chromium (Cr)	0.51 ± 0.06	0.62 ± 0.06
Copper (Cu)	2.92 ± 0.15	5.03 ± 0.94
Iron (Fe)	463.33 ± 7.23	489.73 ± 7.67
Potassium (K)	106.55 ± 4.80	59.74 ± 2.17
Magnesium (Mg)	150.20 ± 9.73	96.10 ± 7.03
Manganese (Mn)	129.66 ± 4.65	96.85 ± 6.11
Sodium (Na)	7.53 ± 1.61	15.03 ± 1.74
Nickel (Ni)	0.41 ± 0.05	0.78 ± 0.06
Phosphorus (P)	31.20 ± 1.59	25.36 ± 1.16
Lead (Pb)	2.07 ± 0.17	1.12 ± 0.11
Zinc (Zn)	3.31 ± 0.19	3.36 ± 0.38
